# Comparison of different anticoagulation methods in continuous renal replacement therapy for pediatric acute liver failure patients: a retrospective observational study

**DOI:** 10.3389/fped.2025.1667760

**Published:** 2025-08-26

**Authors:** Jie He, Xinping Zhang

**Affiliations:** Pediatric Intensive Care Unit, The Affiliated Children’s Hospital of Xiangya School of Medicine, Central South University/Hunan Children’s Hospital, Changsha, China

**Keywords:** acute liver failure, continuous renal replacement therapy, children, anticoagulant, heparin, regional citrate anticoagulation, nafamostat mesylate

## Abstract

**Objective:**

Optimal anticoagulation for pediatric acute liver failure (ALF) patients requiring continuous renal replacement therapy (CRRT) remains challenging due to concurrent bleeding risk and hypercoagulability. This study aimed to evaluate the efficacy and safety of various anticoagulation strategies in pediatric ALF.

**Methods:**

We retrospectively analyzed 51 children with ALF from January 2017 to December 2023. Patients were grouped based on anticoagulant: systemic heparin anticoagulation group (SHA group, *n* = 19), regional citrate anticoagulation group (RCA group, *n* = 15), and nafamostat mesylate group (NM group, *n* = 17). Primary outcomes were filter lifespan and the incidence of new clinical bleeding episodes.

**Results:**

Filter lifespan was shortest in the SHA group but similar between the RCA and NM groups [SHA: 37.0 [34.0, 42.0] h; RCA: 43.0 [39.0, 49.0] h; NM: 43.0 [40.5, 48.0] h; *P* = 0.003]. The SHA group experienced a significantly higher rate of new bleeding episodes (36.8%) compared with the RCA (6.7%) and NM (5.9%) groups (*P* = 0.036). Metabolic alkalosis and hypocalcemia were more frequent in the RCA group (46.7% vs. 10.5% vs. 11.8%; *P* < 0.001). Multivariate Cox regression showed that, relative to SHA, both RCA and NM significantly reduced filter clotting risk (HR = 0.108, 95% CI 0.047–0.248, *P* < 0.001). Additionally, higher pre-CRRT platelet count (HR = 1.014, 95% CI 1.007–1.021, *P* < 0.001), and higher initial transmembrane pressure (HR = 1.168, 95% CI 1.104 −1.236, *P* < 0.001) were associated with increased clotting risk.

**Conclusion:**

In ALF children undergoing CRRT, both RCA and NM demonstrate superior filter longevity and bleeding safety compared to heparin. NM may be preferred due to fewer metabolic disturbances than RCA.

## 1 Introduction

Pediatric acute liver failure (PALF) exhibits a high case fatality rate, ranging from 11% to 67.6% (1–3), and conventional treatments often prove insufficient to improve prognosis effectively. Continuous renal replacement therapy (CRRT) facilitates metabolic clearance and fluid management, thereby providing critical time for liver regeneration or liver transplantation (4), and ultimately enhancing overall survival and transplant-free survival rates (5). Anticoagulation therapy is essential for the successful operation of CRRT. However, in patients with acute liver failure (ALF), the simultaneous reduction of both procoagulant and anticoagulant factors creates a paradoxical state characterized by high bleeding risk concurrent with hypercoagulability. This complex hemostatic imbalance poses substantial challenges to anticoagulation management during CRRT in ALF patients (6, 7). Reported anticoagulation methods for CRRT include systemic heparin anticoagulation (SHA), regional citrate anticoagulation (RCA), low-molecular-weight heparin, argatroban, nafamostat mesylate (NM), prostacyclin, and regional heparin, among others (8–13). Currently, the mainstream anticoagulation protocols are SHA and RCA (14). The use of SHA in ALF is limited by its inherent bleeding risk (15). Although RCA can extend filter lifespan and is associated with a lower bleeding risk (11, 14), its reliance on liver metabolism was once considered a contraindication in liver failure. Nonetheless, recent studies suggest that, with meticulous monitoring, RCA can be safely employed in liver failure (16–18), although supporting clinical evidence remains insufficient. As a novel serine protease inhibitor, nafamostat mesylate (NM) demonstrates unique advantages (19), through a multi-target anticoagulation mechanism that includes inhibition of factors IIa, Xa, and XIIa as well as platelet activation. NM is rapidly degraded in the blood by carboxylesterase, an enzyme highly expressed in metabolic organs such as the liver, intestines, and kidneys (20). With a molecular weight of only 539 Da, below the retention threshold of conventional filters, and a plasma half-life of merely 8 min, NM serves as an effective regional anticoagulant in extracorporeal settings. Theoretically, even patients with liver failure can metabolize and clear NM. To date, no literature has reported on the use of NM in CRRT for liver failure patients. The selection of anticoagulation strategies for CRRT in PALF remains controversial, and systematic comparisons of the efficacy and safety of SHA, RCA, and NM are lacking. This study aimed to determine an optimal anticoagulation strategy for PALF patients undergoing CRRT by conducting a clinical cohort analysis to evaluate the overall benefits of these three protocols for the first time.

## 2 Materials and methods

### 2.1 Study participants

This retrospective observational single-center study was conducted to analyze data from patients with PALF who underwent CRRT in the Pediatric Intensive Care Unit of Hunan Children's Hospital from January 2017 to December 2023. Inclusion criteria were: (1) a PALF diagnosis meeting the Squires JE criteria (21); (2) CRRT performed using continuous veno-venous hemodiafiltration (CVVHDF); and (3) exclusive use of SHA, RCA, or NM as the anticoagulation method. Exclusion criteria included: (1) discontinuation of CRRT for non-circuit reasons (e.g., discharge due to withdrawal of care or death); (2) patients who left the unit for examinations or other non-circuit-related reasons during CRRT; and (3) any instance where arterial oxygen partial pressure was less than 60 mmHg under any form of oxygenation. Study participants were categorized into three groups based on the anticoagulation method employed during CRRT: the SHA, RCA, and NM groups. Pediatric End-Stage Liver Disease (PELD)/Model for End-Stage Liver Disease (MELD) scores ≥28 were associated with poor prognosis (22). Based on this, we stratified children in the RCA group into high-risk (score >28) and non-high-risk (score ≤28) subgroups using 28 as the cut-off value. Regardless of the number of CRRT sessions a patient underwent in the pediatric intensive care unit (PICU), only the first session was analyzed. This study was approved by the Ethics Committee of Hunan Children's Hospital, approval number [HCHLL-2025-37]. The study was conducted in accordance with the ethical principles of the Declaration of Helsinki. Due to the retrospective nature of the study and the use of previously collected medical records, obtaining written informed consent from participants’ legal guardians was not feasible. The Ethics Committee granted a waiver of informed consent in accordance with ethical guidelines. All patient data were anonymized to maintain confidentiality, and no identifying information is included in this manuscript.

### 2.2 CRRT prescription

The initiation of CRRT was determined by the attending physician in the PICU based on the clinical condition of the child. CVVHDF was used as the primary CRRT mode. The choice of the anticoagulation method for CRRT was also determined by the attending physician in the PICU according to the condition of the child. CRRT was performed through either Prismaflex devices (Gambro, Lund, Sweden) or the multiFiltrate multifunctional blood purification system (Fresenius Medical Care, Bad Homburg, Germany), along with the corresponding tubing and filters. Vascular access was established under ultrasound guidance, preferably through the right internal jugular or femoral vein, typically with a single-lumen, double-cuffed catheter. During the treatment, the child's blood gas analysis, complete blood count, liver and kidney function, blood electrolytes, and coagulation function were closely monitored. CRRT treatment parameters were dynamically adjusted on the basis of the patient's clinical presentation and monitoring results.

### 2.3 CRRT anticoagulation strategy

Patients receiving anticoagulation with sodium heparin injection were categorized into the SHA group. The heparin loading dose was 10–50 U/kg administered via intravenous bolus, followed by a maintenance dose of 5–20 U/(kg•h) delivered through continuous intravenous infusion. At the end of treatment, protamine was administered to neutralize the heparin. During CRRT, if the activated partial thromboplastin time (APTT) exceeded twice the normal value or the activated clotting time (ACT) was greater than 180 s, the heparin dose was reduced. If the patient developed new clinical bleeding episodes, the heparin dose was either reduced or discontinued.

Patients receiving anticoagulation with 4% trisodium citrate solution only were categorized into the RCA group (Chengdu Qingshan Likang Pharmaceutical Co., Ltd., China). The initial citrate flow rate (ml/h) was calculated as 1.5 × the blood pump flow rate (ml/min). The target ionized calcium level was 1–1.2 mmol/L for systemic circulation (*in vivo*) and 0.2–0.4 mmol/L for the extracorporeal circuit (*in vitro*).

Patients receiving anticoagulation with nafamostat mesylate injection (Jiangsu Durui Pharmaceutical Co., Ltd.) were categorized into the NM group. A total of 20 mg of nafamostat mesylate was dissolved in 2–5 ml of 5% glucose injection and then added to 500 ml of 0.9% sodium chloride solution to prime the extracorporeal circuit. The initial dose was 0.1–0.5 mg/kg, and the maintenance dose was 0.1–0.5 mg/(kg•h). The target APTT was set at 1.5–2.5 times the baseline value, or the ACT was maintained between 150 and 250 s. The NM dose was adjusted based on monitoring results.

### 2.4 Data collection

Demographic characteristics, clinical data, CRRT-related parameters, and outcomes of the study participants were documented. The primary endpoints included filter lifespan and the occurrence of new clinical bleeding episodes, serving to assess the effectiveness and safety of anticoagulation during CRRT. The secondary endpoints comprised new metabolic abnormalities related to anticoagulant metabolism or the exacerbation of preexisting metabolic disorders during CRRT. Key metabolic abnormalities of interest were citrate accumulation, metabolic acidosis, metabolic alkalosis, hypocalcemia, hypercalcemia, hypernatremia, hyperkalemia, and hyponatremia.

### 2.5 Statistical methods

Data analysis was conducted using SPSS statistical software version 25.0. Categorical data were expressed as absolute counts (percentages) and compared using the *χ*^2^ test. For non-normally distributed continuous variables, the median (interquartile range) [M (P25, P75)] was effectively utilized, and the Kruskal–Wallis H test was applied for group comparisons. Filter survival rates were evaluated via the cumulative incidence function and the Fine-Gray test, with filter failure (i.e., functional death requiring replacement) regarded as a competing risk factor. Factors influencing filter clotting were analyzed using Cox proportional hazards regression. First, univariate Cox regression identified 5 statistically significant variables (*P* < 0.05). For time-varying events such as red blood cell transfusion and plasma transfusion, the proportional hazards assumption was assessed via Schoenfeld residual plots, with a global test *P*-value >0.05 confirming compliance with proportional hazards assumptions. Consequently, these 5 variables were included in the multivariable Cox proportional hazards model to calculate hazard ratios (HR) and 95% confidence intervals (95% CI). A significance level of *P* < 0.05 was applied to all statistical tests. Data visualization was performed using the R language, version 4.4.2 (Integrated Development for R. RStudio, Inc., Boston, Massachusetts, USA).

## 3 Results

### 3.1 General characteristics of study participants

**Figure 1:**
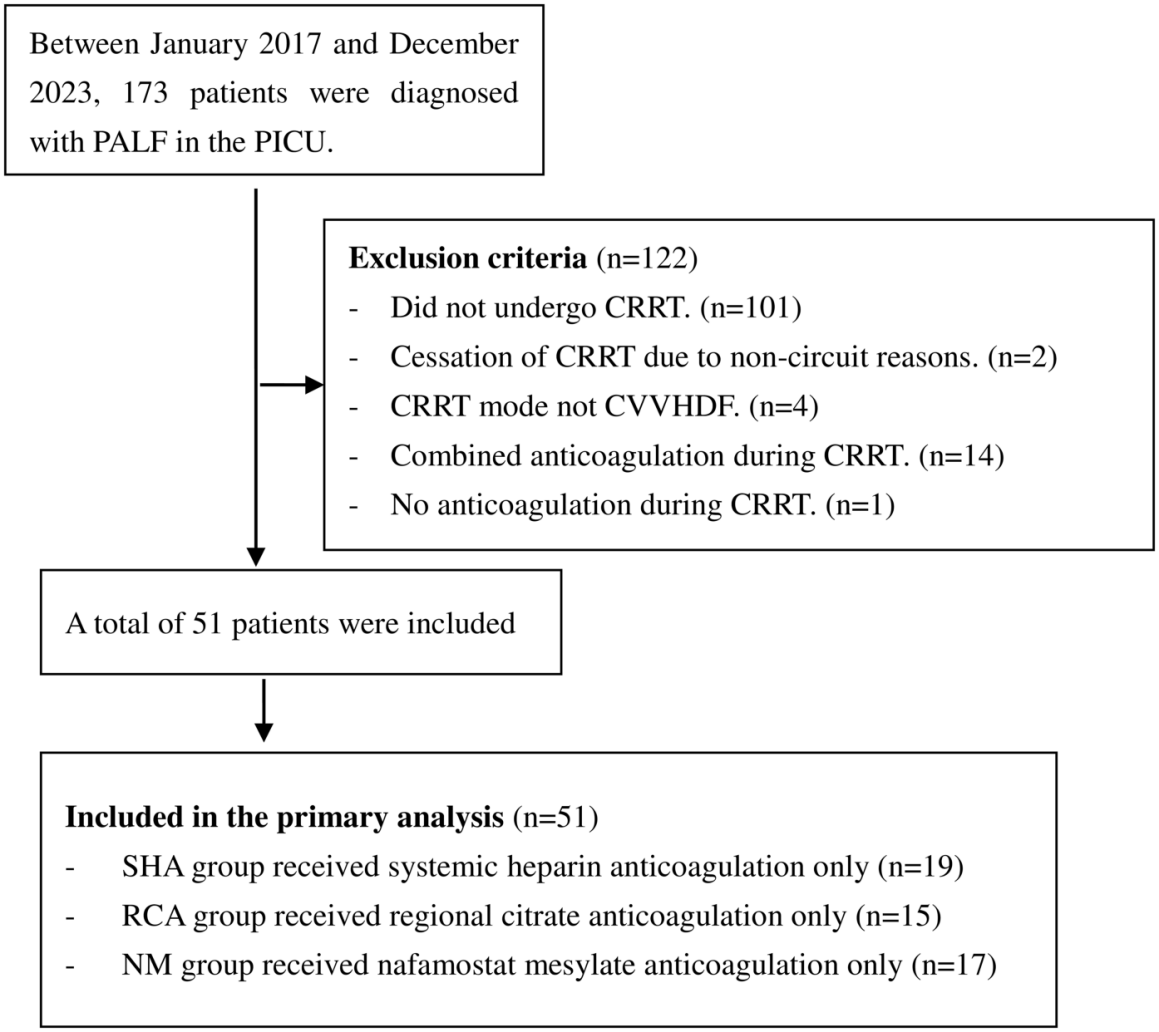
Flowchart of the patient selection process. PALF, pediatric acute liver failure; PICU, pediatric intensive care unit; CRRT, continuous renal replacement therapy; CVVHDF, continuous veno-venous hemodiafiltration; SHA, systemic heparin anticoagulation; RCA, regional citrate anticoagulation; NM, nafamostat mesylate.

A total of 51 children were analyzed on the basis of the inclusion and exclusion criteria. The complete study participants’ selection process is clearly illustrated in Figure 1. Among them, there were 19 patients in the SHA group, 15 in the RCA group, and 17 in the NM group. Before CRRT, 86.3% of the children required mechanical ventilation for respiratory support, and 68.6% required vasopressor support. All children in the three groups exhibited coagulation dysfunction, characterized by prolonged PT, APTT, and INR, decreased platelet counts, and elevated D-dimer levels, presenting a paradoxical state of both high bleeding risk and hypercoagulability. The causes of PALF were as follows: viral hepatitis (*n* = 10), toxic hepatitis (*n* = 10), sepsis (*n* = 9), unknown (*n* = 9), Wilson disease (*n* = 8), drug-induced hepatitis (*n* = 3), and metabolic disease (*n* = 2). The baseline characteristics were comparable between the three groups, as shown in Table 1.

**Table 1:** Baseline characteristics of study participants before initiation of CRRT.

Indicators	SHA group	RCA group	NM group	*P*-value
Number of patients	19	15	17	
Age in months [*M (P25, P75),* months]^a^	56.0 (12.0, 141.0)	53.0 (23.0, 96.0)	51.0 (6.0, 115.5)	0.614
Gender (male/female, n)^b^	10/9	9/5	8/9	0.721
Weight [*M (P25, P75),* kg]^a^	19.5 (12.3, 30.0)	18.0 (11.0, 30.7)	15.0 (7.0, 34.5)	0.738
MELD/PELD score [*M (P25, P75)]*^a^	23.0 (17.0, 26.0)	23.0 (19.0, 32.0)	24.0 (20.5, 27.0)	0.591
Mechanical ventilation [*n*(%)]^b^	16 (84.2)	13 (86.7)	15 (88.2)	1.000
Vasopressor use [*n*(%)]^b^	10 (52.6)	12 (80.0)	13 (76.5)	0.197
Prothrombin time [*M (P25, P75)*, s]^a^	25.8 (23.7, 29.5)	27.8 (24.8, 36.2)	26.1 (24.2, 28.8)	0.374
APTT [*M (P25, P75)*, s]^a^	58.9 (54.3, 72.3)	67.2 (57.9, 76.2)	63.2 (55.8, 66.4)	0.497
INR [*M (P25, P75)]*^a^	2.44 (2.18, 2.90)	2.69 (2.31, 3.79)	2.47 (2.23, 2.81)	0.389
D-dimer level [*M (P25, P75)*, μg/ml]^a^	1.28 (0.78, 3.31)	1.45 (0.82, 4.27)	1.98 (0.81, 4.73)	0.812
Platelet (*M [P25, P75]*, ×10^9 ^/L)^a^	59.0 (40.0, 138.0)	35.0 (29.0, 109.0)	34.0 (26.5, 61.0)	0.135
Hemoglobin [*M (P25, P75)*, g/L]^a^	83.0 (73.0, 88.0)	79.0 (75.0, 87.0)	84.0 (74.5, 86.5)	0.917
pH [*M (P25, P75)*]^a^	7.24 (7.21,7.34)	7.24 (7.17,7.31)	7.25 (7.20,7.32)	0.976
Total bilirubin [*M (P25, P75)*, mmol/L]^a^	200.3 (124.7, 260.1)	148.7 (123.6, 306.9)	186.8 (119.4, 304.4)	0.984
Direct bilirubin [*M (P25, P75)*, mmol/L]^a^	114.6 (70.3, 143.3)	84.7 (68.5, 196.2)	119.5 (68.5, 186.7)	0.920
ALT [*M (P25, P75)*, IU/L]^a^	960.1 (304.3, 1,502.9)	1,049.8 (523.6, 1,722.6)	757.3 (472.5, 1,507.6)	0.698
AST [*M (P25, P75)*, IU/L]^a^	740.8 (421.4, 1,231.8)	962.8 (393.8, 1,452.7)	1,079.0 (515.8, 1,485.3)	0.720
Serum albumin [*M (P25, P75)*, g/L]^a^	28.4 (27.1, 31.1)	28.6 (26.1, 30.8)	27.9 (24.5, 30.0)	0.541
Serum ammonia [*M (P25, P75)*, μmol/L]^a^	49.8 (33.8, 76.3)	33.3 (23.3, 91.6)	48.6 (32.0, 61.9)	0.454
Serum lactic acid [*M (P25, P75)*, mmol/L]^a^	2.5 (2.3, 3.6)	2.5 (1.8, 2.8)	2.7 (2.2, 3.3)	0.299
HCO^−^₃ [*M (P25, P75)*, mmol/L]^a^	21.3 (15.8,24.3)	21.1 (17.9,24.7)	17.1 (15.2,21.1)	0.113
Blood urea nitrogen [*M (P25, P75)*, mmol/L]^a^	6.9 (6.4, 8.0)	7.1 (6.2, 9.0)	7.9 (6.8, 9.4)	0.334
Serum creatinine [*M (P25, P75)*, μmol/L]^a^	73.2 (61.4, 83.5)	92.6 (72.5, 118.3)	85.4 (69.3, 94.3)	0.117
Serum calcium [*M (P25, P75)*, mmol/L]^a^	2.36 (2.19,2.54)	2.32 (2.22,2.50)	2.39 (2.23,2.54)	0.880
Systemic ionized calcium [*M (P25, P75)*, mmol/L]^a^	1.22 (1.08,1.26)	1.25 (1.14,1.31)	1.23 (1.10,1.30)	0.464
Serum sodium [*M (P25, P75)*, mmol/L]^a^	137.3 (133.2,139.9)	134.8 (132.4,138.7)	135.3 (133.2,137.9)	0.503
Serum potassium [*M (P25, P75)*, mmol/L]^a^	4.0 (3.8, 4.3)	3.9 (3.4, 4.4)	4.5 (3.8, 4.7)	0.182
Etiology of PALF (*n*/%)^b^				0.941
Viral hepatitis (*n*/%)	2 (10.5)	5 (33.3)	3 (17.6)	
Sepsis (*n*/%)	3 (15.8)	2 (13.3)	4 (23.5)	
Toxic hepatitis (*n*/%)	4 (21.1)	3 (20.0)	3 (17.6)	
Drug-induced hepatitis (*n*/%)	1 (5.3)	1 (6.7)	1 (5.9)	
Metabolic disease (*n*/%)	1 (5.3)	0 (0)	1 (5.9)	
Wilson disease (*n*/%)	3 (15.8)	3 (20.0)	2 (11.8)	
Unknown (*n*/%)	5 (26.3)	1 (6.7)	3 (17.6)	
CRRT indication(*n*/%)^b^				0.929
Hyperammonemia (*n*/%)	2 (10.5)	3 (20.0)	2 (11.8)	
Acute kidney injury (*n*/%)	5 (26.3)	4 (26.7)	3 (17.6)	
Volume overload (*n*/%)	4 (21.1)	3 (20.0)	6 (35.3)	
Other (*n*/%)	8 (42.1)	5 (33.3)	6 (35.3)	

MELD/PELD, Model for End-Stage Liver Disease/Pediatric End-Stage Liver Disease; APTT, activated partial thromboplastin time; INR, international normalized ratio; ALT, alanine aminotransferase; AST, aspartate aminotransferase; PALF, pediatric acute liver failure.

^a^ Kruskal–Wallis H test;

^b^ Chi-square test or Fisher's exact test.

### 3.2 Filter lifespan and bleeding episodes

**Figure 2:**
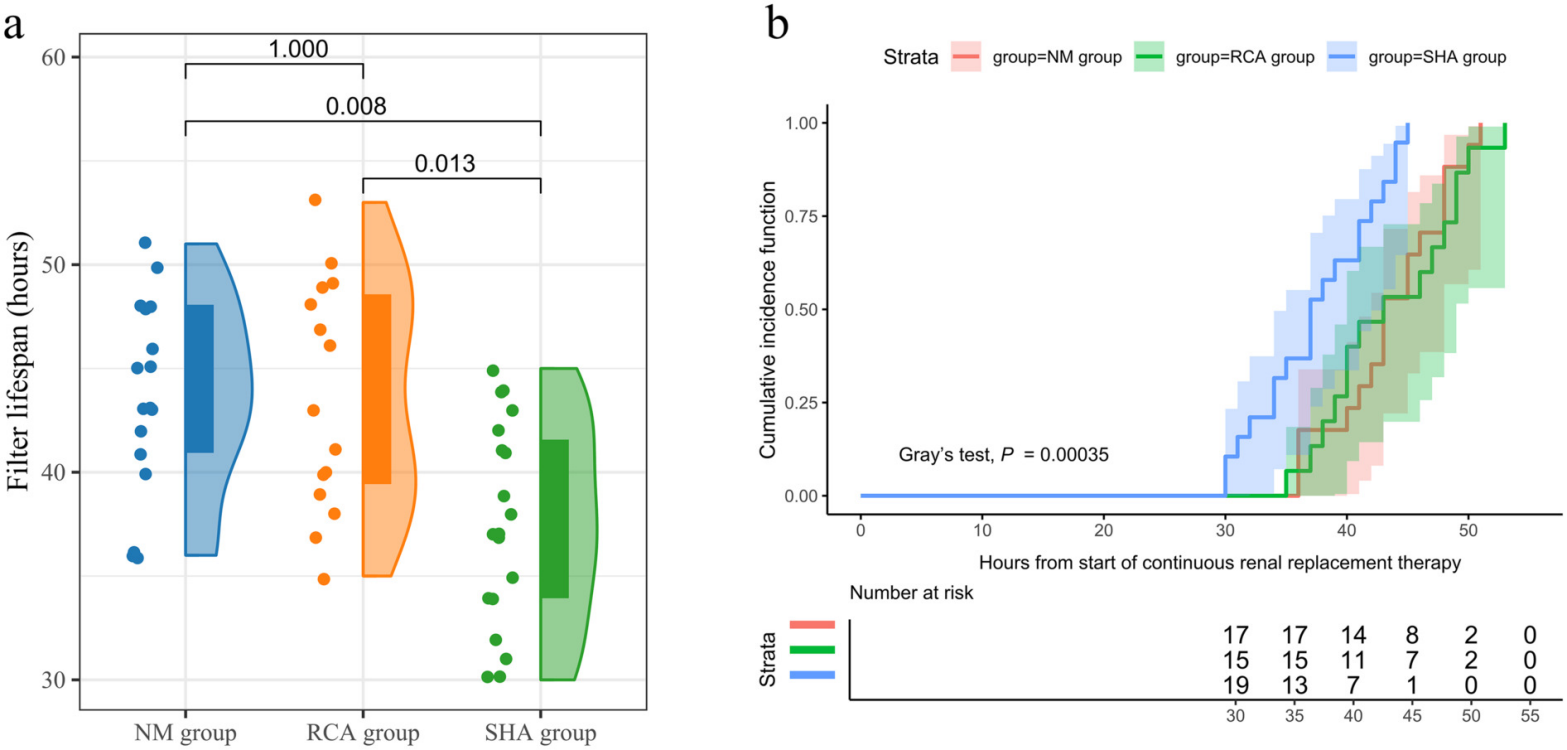
Filter lifespan. **(a)** Filter lifespan in the SHA, RCA, and NM groups depicted as a raincloud plot with median and interquartile range. **(b)** Cumulative incidence function curves illustrating filter survival across the SHA, RCA, and NM groups. Filter failure (i.e., functional death requiring replacement) is regarded as a competing risk factor.

Comparative analysis revealed that the SHA group exhibited the shortest filter lifespan, whereas the RCA and NM groups demonstrated similar durations [SHA group: 37.0 [34.0, 42.0] h; RCA group: 43.0 [39.0, 49.0] h; NM group: 43.0 [40.5, 48.0] h, *P* = 0.003] (Figure 2A). The cumulative incidence function analysis for filter failure indicated that no failures occurred in any group during the first 24 h of treatment. By 36 h, the failure rate in the SHA group increased to 36.84%, compared to 6.67% in the RCA group and 5.88% in the NM group. By 48 h, the cumulative failure rate in the SHA group reached 100%, while the RCA and NM groups showed rates of 73.33% and 76.47%, respectively. Additionally, filters in the SHA group failed entirely within 45 h, in contrast to 53 h and 51 h for the RCA and NM groups, respectively, reflecting more sustained treatment continuity. Figure 2B displays the cumulative incidence function of filter survival rates.

The incidence of new clinical bleeding episodes was significantly higher in the SHA group than that in the RCA and NM groups (36.8% vs. 6.7% vs. 5.9%, *P* = 0.036), with no statistically significant difference between the RCA and NM groups (*P* = 0.932). Moreover, the incidence of red blood cell transfusion during CRRT trended higher in the SHA group (42.1% vs. 13.3% vs. 11.7%), though the difference did not reach statistical significance (*P* = 0.072). CRRT-related indicators and outcomes are detailed in Table 2.

**Table 2:** CRRT-Related indicators and outcomes.

Indicators	SHA group	RCA group	NM group	*P*-value
Number of patients	19	15	17	
RBC transfusion during CRRT [*n* (%)]^g^	8 (42.1)	2 (13.3)	2 (11.7)	0.072
Plasma transfusion during CRRT [*n* (%)]^g^	3 (15.8)	1 (6.7)	1 (5.9)	0.606
Pump flow rate [*M(P25,P75),*ml/min]^a^^,^^f^	4.9 (4.6, 5.0)	3.0 (2.8, 3.0)	5.0 (4.1, 5.0)	<0.001
Effluent rate [*M (P25, P75)*, ml/kg/h]^a^^,^^f^	31.5 (30.1, 32.7)	41.4 (40.5, 42.4)	32.1 (30.6, 33.0)	<0.001
Initial Transmembrane Pressure [*M (P25, P75)*, mmHg]^f^	51.0 (39.0, 61.0)	49.0 (43.0, 59.0)	41.0 (39.5, 56.0)	0.312
Maximum Transmembrane Pressure [*M (P25, P75)*, mmHg]^b^^,^^f^	178.0 (113.0, 208.0)	112.0 (81.0, 161.0)	104.0 (90.5, 139.5)	0.012
End-of-Treatment TMP [*M (P25, P75)*, mmHg]^c^^,^^f^	160.0 (97.0, 199.0)	93.0 (76.0, 141.0)	91.0 (82.0, 114.0)	0.006
Catheter location^g^				0.946
Right internal jugular vein [*n* (%)]	8 (42.1)	7 (46.7)	8 (47.1)	
Femoral vein [*n* (%)]	11 (57.9)	8 (53.3)	9 (52.9)	
Filter lifespan [*M (P25, P75)*, h]^d^^,^^f^	37.0 (34.0, 42.0)	43.0 (39.0, 49.0)	43.0 (40.5, 48.0)	0.003
Bleeding episodes [*n* (%)]^e^^,^^f^	7 (36.8)	1 (6.7)	1 (5.9)	0.036
ICU stay [*M (P25, P75), d*]^f^	15.0 (12.0, 17.0)	13.0 (7.0, 15.0)	14.0 (9.0, 16.5)	0.280
ICU mortality [*n* (%)]^g^	6 (31.6)	5 (33.3)	5 (29.4)	1.000
28-d mortality [*n* (%)]^g^	7 (36.8)	5 (33.3)	6 (35.3)	1.000

^a^ The SHA group had a *p*-value of 1.000 when compared to the NM group, while the RCA group had *p*-values of less than 0.001 when compared to both the SHA and NM groups.

^b^ The RCA group had a *p*-value of 1.000 when compared to the NM group and a *p*-value of 0.034 when compared to the SHA group, and the NM group had a *p*-value of 0.031 when compared to the SHA group.

^c^ The RCA group had a *p*-value of 1.000 when compared to the NM group and a *p*-value of 0.016 when compared to the SHA group, and the NM group had a *p*-value of 0.022 when compared to the SHA group.

^d^ The RCA group had a *p*-value of 1.000 when compared to the NM group and a *p*-value of 0.013 when compared to the SHA group, and the NM group had a *p*-value of 0.008 when compared to the SHA group.

^e^ The RCA group had a *p*-value greater than 0.05 when compared to the NM group, while the SHA group had *p*-values of less than 0.05 when compared to both the RCA and NM groups.

^f^ Kruskal–Wallis H test;

^g^ Chi-square test or Fisher's exact test.

### 3.3 Metabolic complications

The incidences of metabolic alkalosis (46.7% vs. 10.5% vs. 11.8%) and hypocalcemia (46.7% vs. 10.5% vs. 11.8%) in the RCA group were significantly higher than those in the SHA and NM groups (*P* < 0.001). None of these metabolic abnormalities manifested clinically significant symptoms, interrupted CRRT therapy, and could be ameliorated by adjusting treatment parameters. The incidence of other metabolic complications showed no statistically significant differences among the three groups (Table 3). In the comparative analysis between high-risk and low-risk subgroups of RCA-treated children, no statistically significant differences were observed in complication rates. See Table 4 for details.

**Table 3:** Metabolic complications.

Indicators	SHA group	RCA group	NM group	*P*-value
Number of patients	19	15	17	
Metabolic alkalosis (*n*/%)	2 (10.5)	7 (46.7)	2 (11.8)	0.031
Metabolic acidosis (*n*/%)	2 (10.5)	1 (6.7)	1 (5.9)	1.000
Citrate accumulation (*n*/%)	0 (0)	0 (0)	0 (0)	1.000
Hypocalcemia (*n*/%)	2 (10.5)	7 (46.7)	2 (11.8)	0.031
Hypercalcemia (*n*/%)	1 (5.3)	1 (6.7)	1 (5.9)	1.000
Hypernatremia (*n*/%)	0 (0)	0 (0)	0 (0)	1.000
Hyponatremia (*n*/%)	3 (15.8)	2 (2.9)	2 (11.8)	1.000
Hyperkalemia (*n*/%)	0 (0)	0 (0)	0 (0)	1.000
Hypokalemia (*n*/%)	1 (5.3)	0 (0)	0 (0)	1.000

All data in Table 3 were analyzed using Fisher's exact test.

**Table 4:** Comparison of metabolic complications between subgroups in the RCA group.

Indicators	High-risk subgroup	Non-high-risk subgroup	*P* value
Number of patients	5	10	
Metabolic alkalosis (*n*/%)	3 (60.0)	4 (40.0)	0.608
Metabolic acidosis (*n*/%)	0 (0)	1 (10.0)	1.000
Citrate accumulation (*n*/%)	0 (0)	0 (0)	1.000
Hypocalcemia (*n*/%)	2 (40.0)	5 (50.0)	1.000
Hypercalcemia (*n*/%)	1 (20.0)	0 (0)	0.333
Hypernatremia (*n*/%)	0 (0)	0 (0)	1.000

All data in Table 4 were analyzed using Fisher's exact test.

### 3.4 Potential factors associated with the risk of filter clotting

Univariate and multivariate Cox regression analyses demonstrated that the anticoagulation method, red blood cell transfusion during CRRT, pre-CRRT platelet count, plasma transfusion during CRRT, and initial transmembrane pressure (TMP) were all independent factors influencing the risk of filter clotting. Among these, RCA or NM anticoagulation served as independent protective factors, while the other five variables were identified as independent risk factors. Specifically, compared to SHA, RCA, or NM anticoagulation significantly reduced the risk of filter clotting (HR = 0.108, 95% CI 0.047–0.248, *P* < 0.001). Additionally, higher pre-CRRT platelet count [HR = 1.014, 95% CI (1.007–1.021), *P* < 0.001] and higher initial TMP (HR = 1.168, 95% CI 1.104–1.236, *P* < 0.001) were all significantly associated with an increased risk of filter clotting (Table 5).

**Table 5:** Cox regression analysis of factors affecting filter lifespan.

Variable	Univariate analysis		Multivariate analysis	
	HR (95% CI)	*P*-value	HR (95% CI)	*P*-value
Anticoagulation method	0.579 (0.393, 0.854)	0.006	0.108 (0.047,0.248)	<0.001
Pre-CRRT hemoglobin level	1.012 (0.969, 1.057)	0.578		
RBC transfusion during CRRT	2.988 (1.521, 5.870)	<0.001	1.361 (0.572,3.243)	0.486
Pre-CRRT D-dimer level	1.096 (0.995, 1.209)	0.064		
Pre-CRRT platelet count	1.014 (1.008, 1.019)	<0.001	1.014 (1.007,1.021)	<0.001
Plasma transfusion during CRRT	8.517 (2.803, 25.882)	<0.001	4.079 (0.759,21.918)	0.101
Initial TMP	1.151 (1.102, 1.201)	<0.001	1.168 (1.104,1.236)	<0.001
Pre-CRRT APTT level	1.011 (0.995, 1.027)	0.191		
Pump flow rate	1.218 (0.898, 1.651)	0.205		
Effluent rate	0.957 (0.899, 1.019)	0.169		

## 4 Discussion

To our knowledge, this cohort study is the first to evaluate the effectiveness of SHA, RCA, and NM anticoagulation in CRRT for patients with PALF. The study found that compared to SHA, RCA, or NM anticoagulation significantly reduced the risk of filter clotting during CRRT in patients with PALF and extended the filter lifespan. Additionally, the bleeding risk associated with RCA and NM anticoagulation was significantly lower than that observed with heparin. Further analysis indicated no significant differences between RCA and NM in terms of filter lifespan and bleeding risk, while the incidence of metabolic complications was lower in the NM group compared to the RCA group.

The coagulopathy of PALF is relatively complex (6). Therefore, selecting an appropriate anticoagulation protocol for CRRT in patients with PALF remains a challenge, and no consensus has been reached to date. Heparin is the most commonly used anticoagulant for CRRT in patients with liver failure (17); however, its systemic anticoagulant effects may exacerbate the bleeding risk in patients with PALF. In this study, the incidence of clinical bleeding episodes in the SHA group was significantly higher than that in the RCA and NM groups. Additionally, the SHA group exhibited an increasing trend in red blood cell transfusion requirements, with an approximately 3-fold absolute difference, suggesting potential clinical relevance. In terms of filter efficiency, the median filter lifespan in the SHA group (37.0 h) was significantly shorter than that in the RCA (43.0 h) and NM groups (43.0 h) (*P* = 0.003), further confirming the limitations of systemic anticoagulation in patients with PALF.

The use of RCA in CRRT for patients with liver failure has been controversial. As citrate is primarily metabolized by liver mitochondria, the traditional view suggests that its use in liver failure may lead to citrate accumulation. However, clinical studies have provided new insights into this issue. A prospective multicenter study showed (23) no statistically significant differences in severe acid-base imbalance and calcium metabolism disorders between individuals with normal liver function and those with liver failure. Only 3.5% (3 out of 85) of patients with liver failure exhibited signs of impaired citrate metabolism, suggesting that overall citrate metabolism in patients with liver failure is comparable to that in individuals with normal liver function. A meta-analysis that included 10 studies further confirmed that (24) compared to heparin, RCA can significantly extend the filter lifespan in patients with liver failure, with a citrate accumulation incidence of 12% and severe bleeding episodes reduced to 5%. A randomized controlled trial conducted by Bai et al. (25) demonstrated that, compared to no anticoagulation protocols, RCA in patients with liver failure at high risk of bleeding effectively prolonged filter lifespan without increasing the incidence of clinical bleeding episodes. Although the RCA group showed a higher incidence of hypocalcemia and elevated total calcium/ionized calcium ratios, most cases were improved through dynamic adjustments in calcium supplementation, and no patients exhibited clinical signs of hypocalcemia. There were no statistically significant differences between the groups in key outcome indicators, such as severe metabolic alkalosis, metabolic acidosis, or 28-day all-cause mortality, which aligns with the findings of Fang et al. 18 on pediatric liver failure populations. Another retrospective study further supported these findings (26), showing that during RCA-CRRT in children with acute liver failure, metabolic parameters such as the total calcium/ionized calcium ratio, lactate levels, and arterial blood pH remained stable. Only 10% of patients experienced transient hypocalcemia, and no cases of citrate accumulation were detected. In this study, no citrate accumulation was observed in the children, and the incidence of hypocalcemia was 46.7%, a condition that could also be alleviated by adjusting treatment parameters.

Differences in citrate metabolism-related complications among studies suggest a multidimensional mechanism underlying their occurrence. Beyond the continuous clearance of citrate-calcium complexes by CRRT, residual liver function and the activation of extrahepatic metabolic pathways may play critical roles (27). Extrahepatic organs rich in mitochondria, such as skeletal muscle and renal cortex, also metabolize citrate, indicating that citrate clearance in liver failure is not entirely lost but only partially impaired. Moreover, clinical observations suggest that systemic microcirculatory status may impact citrate metabolism more than traditional liver function indicators (28). Based on existing evidence, the core issue is not absolute contraindications but how to individualize citrate anticoagulation protocols. This includes optimizing citrate infusion rates, adjusting calcium supplementation strategies dynamically, and establishing an early warning system for metabolic complications (16).

Currently, NM is mainly used in clinical settings in East Asia (e.g., Japan, China, and South Korea) (9, 29–32), with its application in CRRT largely based on empirical practices. Kameda et al. (9) reported an average filter lifespan of 26.4 ± 23.4 h for NM anticoagulation during CRRT. This study is the first to report a median filter lifespan of 43.0 h (IQR 40.5–48.0) in the NM group. This result is comparable to that of Miyaji et al. (33) who compared anticoagulants in pediatric CRRT. In that study, the NM group had a median filter lifespan of 38 h (range 22–74), significantly longer than the RCA group's 36 h (range 17–66) (*P* = 0.02). The inhibition of potassium secretion in the renal tubules by NM metabolites increases the risk of hyperkalemia, which is NM's primary pharmacological adverse effect (33). However, no cases of NM-related hyperkalemia during CRRT have been reported in the literature (9, 30). Other potential adverse effects of NM include hyponatremia, allergic reactions, and bone marrow suppression (34). In this cohort study, no new or progressive cases of hyperkalemia were observed in the NM group. Although 2 cases of hyponatremia occurred, no statistically significant differences were found in intergroup comparisons with the SHA and RCA groups. Additionally, no serious adverse events, such as allergic reactions or bone marrow suppression, occurred. Patients with PALF undergoing CRRT experienced fewer metabolic complications in the NM group compared to those in the RCA group. Clinically, existing data suggest that NM offers more controllable safety in CRRT for children with PALF than RCA.

Epoprostenol exerts its anticoagulant effect by inhibiting platelet aggregation, making it theoretically suitable for patients with coagulopathy, such as those with liver disease. A single-center study demonstrated that in children with liver disease undergoing CRRT using epoprostenol anticoagulation, the median filter lifespan reached 48 (IQR 32–72) hours, with a 60 h effective filter survival rate of 60.5%. The risks of bleeding and hypotension were similar to those reported with other anticoagulants. (10) However, current reports on its use in pediatric CRRT are very limited; its precise efficacy and safety require validation through prospective studies. Bivalirudin is a direct thrombin inhibitor. Currently, there are reports of bivalirudin use in children on extracorporeal membrane oxygenation (35), but studies investigating its use exclusively for CRRT in children are lacking.

Heparin demonstrated the lowest economic cost with high availability. In contrast, sodium citrate solution incurred the highest cost, further increased by the requirement for synchronized monitoring of both intra- and extracorporeal blood gas parameters. Nafamostat exhibited an intermediate cost, but its application remains geographically restricted primarily to China, Japan, and South Korea, thereby limiting its large-scale adoption.

CRRT filter lifespan is influenced by multiple factors, and previous studies have confirmed that anticoagulation strategies play a crucial role (15, 36, 37) Current evidence indicates that RCA has a significant advantage in extending filter lifespan, outperforming no anticoagulation protocols or SHA (11, 38) Recent studies suggest that the anticoagulant efficacy of NM is not inferior to that of RCA (29) Data show that filter lifespan in both the RCA and NM groups was significantly longer than in the SHA group. Multivariate Cox regression analysis confirmed that using RCA or NM significantly reduces the risk of filter clotting compared to SHA. The multivariate Cox regression analysis in this study revealed that a higher pre-CRRT platelet count was associated with an increased risk of filter clotting, consistent with the findings from a study involving 1,332 CRRT sessions in critically ill adults, which demonstrated that a lower platelet count was strongly associated with prolonged filter lifespan (39). From a physiological perspective, as platelets are key participants in the coagulation process, we posit that an elevated platelet count significantly increases the probability of mutual platelet aggregation. During CRRT, when blood comes into contact with the filter surface, large numbers of platelets readily adhere rapidly to the membrane, thereby triggering the coagulation cascade. TMP has predictive value for extracorporeal circuit clotting events during CRRT. Each 1 mmHg increase in TMP independently raises the risk of clotting events by 1.5% (95% CI 1.0–2.0, *P* < 0.01) (40). Here, multivariate Cox regression analysis showed that initial significantly impacted filter clotting, further confirming TMP's influence on filter lifespan. Real-time TMP monitoring and timely interventions can enhance treatment continuity.

This study has some limitations. First, as a retrospective observational study, selection bias is a concern. Although statistical matching balanced baseline characteristics, unmeasured confounders may still introduce residual bias. Second, the single-center study design imposed limitations on sample size. Furthermore, the selection of anticoagulation strategy was determined at the discretion of the treating physician based on individual patient factors, potentially introducing selection bias despite comparable baseline characteristics across the three groups. The anticoagulation benefits of NM vs. RCA/SHA require validation through multicenter randomized controlled trials. Third, our center adopted NM anticoagulation in 2020, concentrating data collection in recent years, which may introduce temporal bias and reflect improvements in CRRT management. Lastly, while monitoring protocols for SHA and RCA are well-established, standardized guidelines for NM anticoagulation intensity remain lacking. Currently, NM dose adjustments rely on empirical judgment. This may compromise the accurate efficacy evaluation of NM, whose advantages over RCA still require validation through large-scale prospective studies.

## 5 Conclusion

This study confirms that in children with PALF undergoing CRRT, both RCA and NM anticoagulation significantly outperformed SHA in prolonging filter lifespan. The bleeding risk associated with RCA and NM anticoagulation was also significantly lower than that of heparin. Furthermore, NM was associated with a lower incidence of metabolic complications compared to RCA. Considering both efficacy and safety, NM may be a preferable anticoagulation option for CRRT in children with PALF. However, its clinical utility requires further validation through multicenter randomized controlled trials.

## Data Availability

The original contributions presented in the study are included in the article/Supplementary Material, further inquiries can be directed to the corresponding author.
